# Controlling the Polarity of the Molecular Beam Epitaxy Grown In-Bi Atomic Film on the Si(111) Surface

**DOI:** 10.1038/s41598-018-37051-2

**Published:** 2019-01-24

**Authors:** Cho-Ying Lin, Chia-Hsiu Hsu, Yu-Zhang Huang, Shih-Ching Hsieh, Han-De Chen, Li Huang, Zhi-Quan Huang, Feng-Chuan Chuang, Deng-Sung Lin

**Affiliations:** 1 Department of Physics, National Tsing Hua University, Hsinchu, 30013 Taiwan; 2 Department of Physics, Southern University of Science and Technology, Shenzhen, Guangdong 518055 China; 3 Department of Physics, National Sun Yat-Sen University, Kaohsiung, 804 Taiwan

## Abstract

Synchrotron radiation core-level photoemission spectroscopy, scanning tunneling microscopy (STM), and first-principles calculations have been utilized to explore the growth processes and the atomic structure of the resulting films during the two-step molecular beam epitaxy (MBE) of In and Bi on the Si(111) surface. Deposition of 1.0-ML Bi on the In/Si(111)-(4 × 1) surface at room temperature results in Bi-terminated BiIn-(4 × 3) structures, which are stable up to ~300 °C annealing. By contrast, deposition of In on the β-Bi/Si(111)-(√3 × √3) surface at room temperature results in three dimensional (3D) In islands. In both cases, annealing at 460 °C results in the same In-terminated In_0.75_Bi/Si(111)-(2 × 2) surface. Our DFT calculations confirm that the surface energy of In-terminated In_0.75_Bi/Si(111)-(2 × 2) system is lower than that of Bi-terminated Bi_0.75_In/Si(111)-(2 × 2). These findings provide means for the control of the polarity of the MBE In-Bi atomically thick films.

## Introduction

Two dimensional topological insulators (2D TIs) have attracted much interest in the last decade for their fascinating fundamental physics and possible applications in spintronic and quantum computing devices due to much reduced elastic electron scattering^1–4^. 2D TIs, also known as quantum spin hall insulators, are atomically thin layered materials that exhibit time-symmetry-protected metallic edge states with an insulating interior^5,6^. Compared to the surface states in the 3D TIs, the edge states in 2D TIs are more robust since the only available backscattering channel is forbidden. In search of new and robust 2D TIs with large band gaps, many possible material systems have been experimentally and theoretically surveyed, including the binary combinations of group III (B, Al, Ga, In, and Tl) and group V (N, P, As, Sb, and Bi) elements in the buckled honeycomb structure^7^.

The III-V compound films on the Si(111) surface are compatible to current silicon-based integrated-circuit technology and have attracted much attention^8–11^. In particular, one or two-bilayer thick films of GaBi and two-bilayer thick films of both pristine and hydrogenated AlBi, GaBi, InBi, TlBi, and TlSb are predicted to be topological insulators with sizable band gaps^8,9^. For the case of In/Bi on Si(111), Denisov *et al*. employed MBE to co-deposit In and Bi with various ratios on the Si(111) surface and following annealing with temperature in the range between 250 and 500 °C. Three type structures of InBi on Si(111) were observed. The atomic models are also simulated to be Bi_0.43_In_0.86_-(√7 × √7), Bi_0.75_In-(2 × 2), Bi_0.48_In_0.56_-(5 × 5)^10^. The (5 × 5) and (√7 × √7) structures are proposed to be one-atomic-layer thick systems, in which the Bi and In atoms reside nearly on the same plane above the bulk-truncated Si(111) substrate. The (2 × 2) structure is modelled to be a Bi-terminated two-atom-layer system with three Bi atoms per (2 × 2) unit cell forming trimer in the top layer and four In atoms in the bottom layer on bulk Si(111).

The lack of inversion symmetry in the <111> directions of III-V compounds gives rise to a difference in various of physical, chemical, and metallurgical properties of the A (group-III terminated) and B (group-V terminated) surfaces^12^. If the substrate effect is insignificant, the differences in the electronic structures and topological properties for the A and B surfaces are small in the 1-bilayer (1BL) system, but become notable in the 2-bilayer (2BL) system with increasing strain^8,9^. For example, the 1BL InBi-Si(111) film possesses a trivial topological insulator phase, while a nontrivial phase is predicted for the 2BL film. It is thus important to distinguish the polarity of the III-V compound film. To address this issue, we employ core-level photoemission spectra, STM, DFT to study the MBE grown InBi-Si(111) system. It is found that the A surface is energetically more favorable, but the B surface can also be obtained in the two-step growth method.

## Experimental Details

The STM measurement was carried out in an UHV chamber with a base pressure below 2.0 × 10^−10^ torr. The Si(111) substrate was cut from silicon wafer with a size of 2 × 10 mm^2^. After 12 hours outgassing at about 500 °C, an atomically clean Si(111) surface was obtained by DC heating to ~1150 °C for a few seconds. The indium and bismuth atomic beams were generated by e-beam evaporators located about 7 cm away from the Si(111) substrate. The deposition rates are ~0.17 ML/min, where 1.0 ML is 7.84 × 10^14^ atoms/cm^2^ for the Si(111) unreconstructed surface.

The photoemission spectra were taken at beamline 24A1 at Taiwan’s National Synchrotron Radiation Research Center from a 1.5 GeV storage ring. The photoelectrons were collected by a 125-mm hemispherical analyzer at take-off angle of ~10° with an acceptance angle of ±8° in a µ-metal shielded UHV chamber, and the overall energy resolution was better than 120 meV with the photon energy of 70 and 130 eV.

The first-principles calculations were carried out within the generalized gradient approximation of the Perdew-Burke-Ernzerhof (PBE) to the density functional theory using projector-augmented-wave (PAW) potentials, as implemented in the Vienna ab-initio simulation package (VASP)^13–17^. The kinetic energy cutoff was set at 250 eV. A periodically repeating slab consisting of four Si bilayers, a reconstructed layer, and a vacuum space of 20 Å was employed. The Si dangling bonds at the bottom of the slab were passivated by hydrogen atoms. Silicon atoms of the bottom bilayer were kept fixed at the bulk crystalline positions corresponding to the theoretical Si lattice constant. In order to obtain optimized atomic positions, the remaining Si, In and Bi atoms were relaxed until the residual force on each atom was smaller than 0.01 eV/Å, which is a general default value in surface calculation for VASP simulation. The criteria for convergence for self-consistent electronic structure set at 0.001 meV, much smaller than the default value 0.1 meV. The Γ-centered 6 × 6 × 1 Monkhorst-Pack grid was used to sample the surface Brillouin-zones (SBZ) for the 2 × 2 phase. Spin-orbit coupling (SOC) was included in band structure calculations^18^.

## Results and Discussion

### DFT calculations for In_0.75_Bi and Bi_0.75_In-(2 × 2) Structures

**Figure 1:**
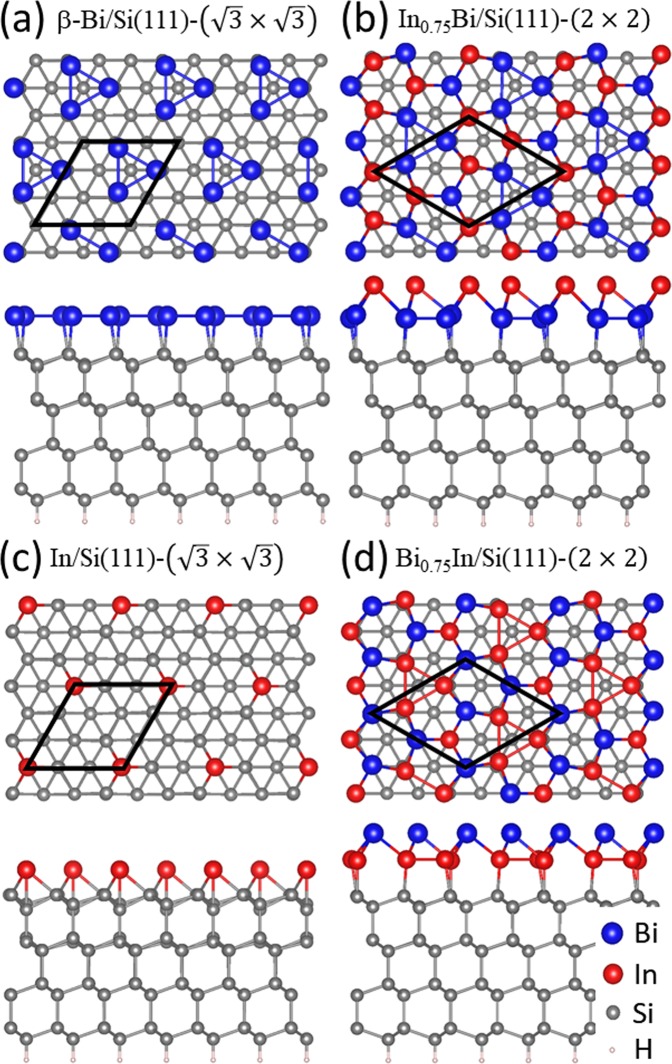
Schematic illustration of the side view for various structures obtained by the DFT calculations. The height difference is 2.28 Å between these two structures of (**a**) and (**b**). The height difference is 2.72 Å between these two structures of (**c**) and (**d**).

In the III-V compound material, the (2 × 2) surface reconstructions for the (111) orientation are the common structures such as GaAs, GaP, GaSb, InAs^19–24^. In the commonly accepted “vacancy buckling model”, each (2 × 2) unit cell on the (111) surface has a missing cation atom, corresponding to a 1/4 monolayer of cation vacancy^25^. Both group-III terminated (A-) surfaces and group-V terminated (B-) surfaces have been found. Denisov *et al*. have observed the similar (2 × 2) reconstruction on Si(111) at 250−500 °C by co-deposition of Bi and In^10^. This process results in the In-rich (2 × 2) phase. They estimated the composition of the (2 × 2)-(Bi, In) structure consisting of 0.68 ± 0.05 ML of Bi and 1.0 ± 0.1 ML of In. A Bi-terminated two-atom-layer system (denoted as Bi_0.75_In/Si) was proposed, in which three Bi atoms per (2 × 2) unit cell on the top outmost layer and four In atoms in the second layer reside on the bulk-like truncated Si(111)-(1 × 1) surface as shown in Fig. 1(d). Our experiments elaborated in the next section show another (2 × 2) phase which is Bi-rich phase. Based on the above mentioned studies, similar vacancy-based model proposed here is that three In atoms per (2 × 2) unit cell on the top outmost layer and four Bi atoms in the second layer reside on the bulk-like truncated Si(111)-(1 × 1) surface as shown in Fig. 1(b). To understand the atomic structures of different growth stages, Fig. 1(a–d) show the atomic superstructures of β-Bi/Si(111)-(√3 × √3), In_0.75_Bi/Si(111)-(2 × 2) (In_0.75_Bi/Si), In/Si(111)-(√3 × √3) and the Bi-terminated Bi_0.75_In/Si(111)-(2 × 2) (denoted as Bi_0.75_In/Si), configurations, respectively.

Our results of calculations for the β-Bi/Si(111)-(√3 × √3) (denoted as β-Bi/Si) and In/Si(111)-(√3 × √3) systems are consistent to those found previously^26–28^. In the β-Bi/Si system, the height difference between Bi and Si top-layer is 2.68 Å, and In_0.75_Bi is 4.93 Å higher than the Si top-layer as shown in Figs 1(a,b). For the two models Bi_0.75_In/Si (Bi-terminated and In-rich model) and In_0.75_Bi/Si (In-terminated and Bi rich model), the relative surface energy Δ*E*_*s*_ is as following,

1 $${\rm{\Delta }}{E}_{s}=(E(I{n}_{0.75}Bi)-E(B{i}_{0.75}In)-{\rm{\Delta }}{N}_{In}{\mu }_{In}-{\rm{\Delta }}{N}_{Bi}{\mu }_{Bi})/A$$

in which the surface area, *A*, of a (2 × 2) supercell is 4 times larger of (1 × 1) unit cell; *μ*_*In*_ and *μ*_*Bi*_ are the chemical potential of In and Bi at bulk phase, respectively. Δ*N*_*In*_ and Δ*N*_*Bi*_ represent the differences in number of In and Bi atoms between two models. The relative surface energy is estimated to Δ*E*_*s*_ = −8.49 meV per (1 × 1) unit cell showing that the In-terminated model has a slightly lower surface energy than that with Bi termination.

### Growth Processes of Bi on the In/Si(111)-(4 × 1) surface

High-resolution X-ray photoemission spectroscopy can distinguish between atoms at nonequivalent sites and in different chemical bonding configurations, based on the atom’s bonding energies (BE). Following standard procedures^29,30^, the spectra were decomposed by least-squares fitting. Identical Voigt line shapes, each consisting of a pair of spin-orbit split (SOS) doublets, were used to decompose the core-level spectra into overlapping components. The fitting parameters are similar for all Si 2*p* spectra: SOS: 0.60 eV; Gaussian full width at half maximum (FWHM): 0.13 eV; Lorentzian width FWHM: 0.37 eV FWHM. Bi 5*d* spectra: SOS: 3.10 eV; Gaussian width FWHM: 0.17 eV; Lorentzian width FWHM: 0.48 eV. In 4*d* spectra SOS: 0.87 eV; Gaussian width FWHM: 0.14 eV; Lorentzian width FWHM: 0.40 eV.

**Figure 2:**
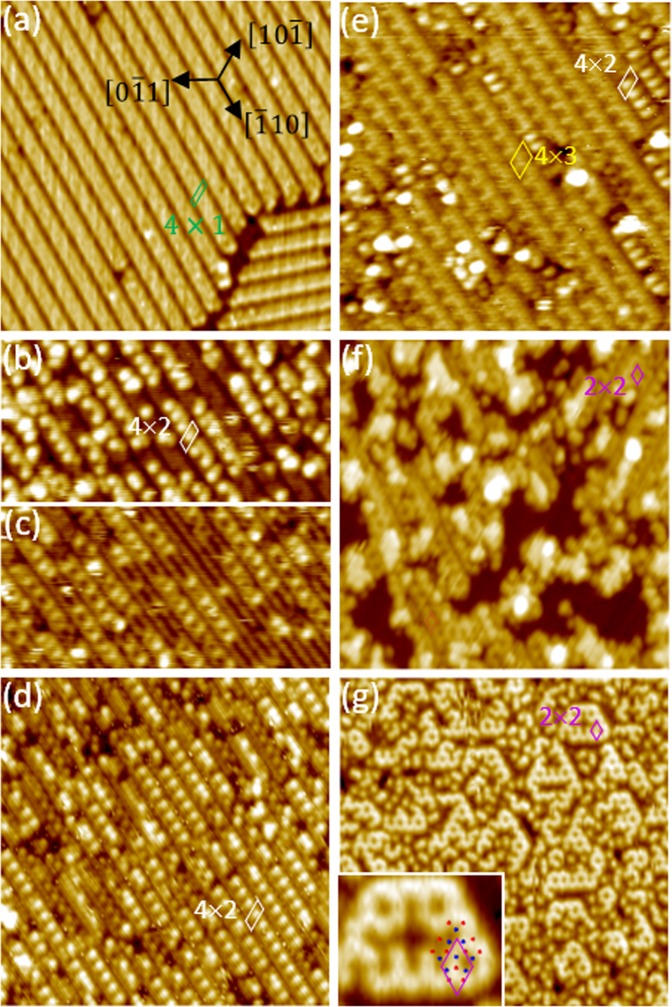
The filled state STM image for (**a**) the In/Si(111)-(4 × 1) surface and the same surface after depositing (**b**) 0.25-, (**d**) 0.50-, and (**e**) 1.0-ML Bi at RT. following by subsequent annealing of the 1.0-ML at temperature of (**f**) 400 °C and (**g**) 460 °C. Image width: 20 nm. V_s_ = −1.9 V; I_t_ = 200 pA; (**c**) empty image for (**b**) V_s_ = 1.9 V. The parallelograms each enclose a unit cell of local ordering as labelled. Inset in (**g**): zoom-in STM image superimposed with the atomic model in Fig. 1(b).

The In/Si(111)-(4 × 1) surface can be formed by the growth of 1.0-ML In on the clean Si(111)^31–39^. Figure 2(a) displays a well-ordered reconstructed (4 × 1) surface obtained by deposition of 1.0 ML In on the clean Si(111)-(7 × 7) surface kept at ~400 °C. The structure of the In/Si(111)-(4 × 1)-In reconstruction has been determined using surface x-ray diffraction (SXRD)^40^, and other techniques^28^. The analysis of SXRD shows that the quasi-one-dimensional character in the image is given by zigzag chains of silicon atoms on top of an unreconstructed silicon substrate and four indium atoms per unit cell (1 ML) arranged in two zigzag chains in the gap between the silicon chains.

**Figure 3:**
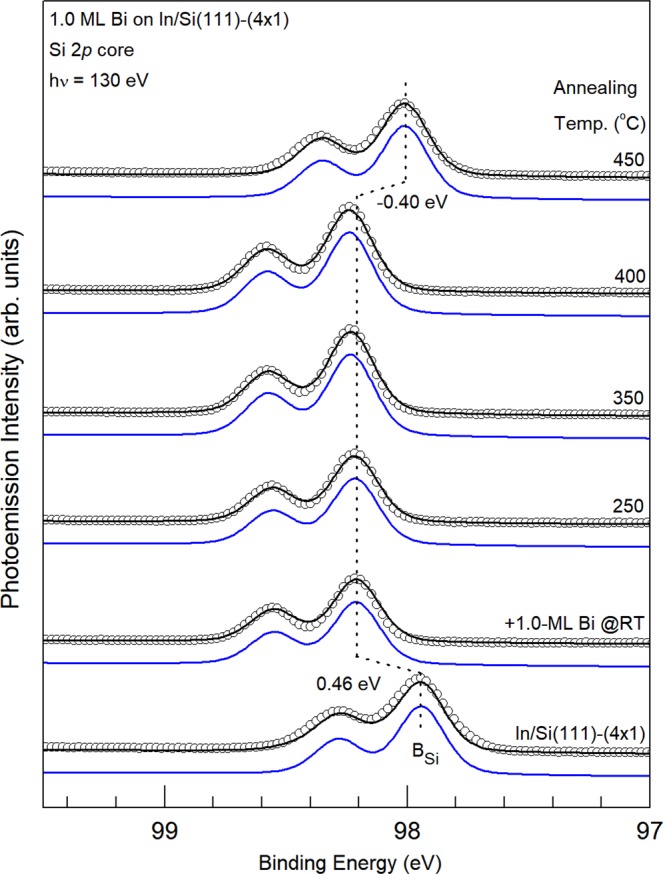
Si 2*p* core level photoemission spectra (circles) for the 1.0-ML In/Si(111)-(4 × 1) surface (bottom), the same surface after 1.0-ML Bi deposited at RT (second to the bottom) and subsequent annealing at various temperature as indicated. Dashed lines are a guide to the eye.

**Figure 4:**
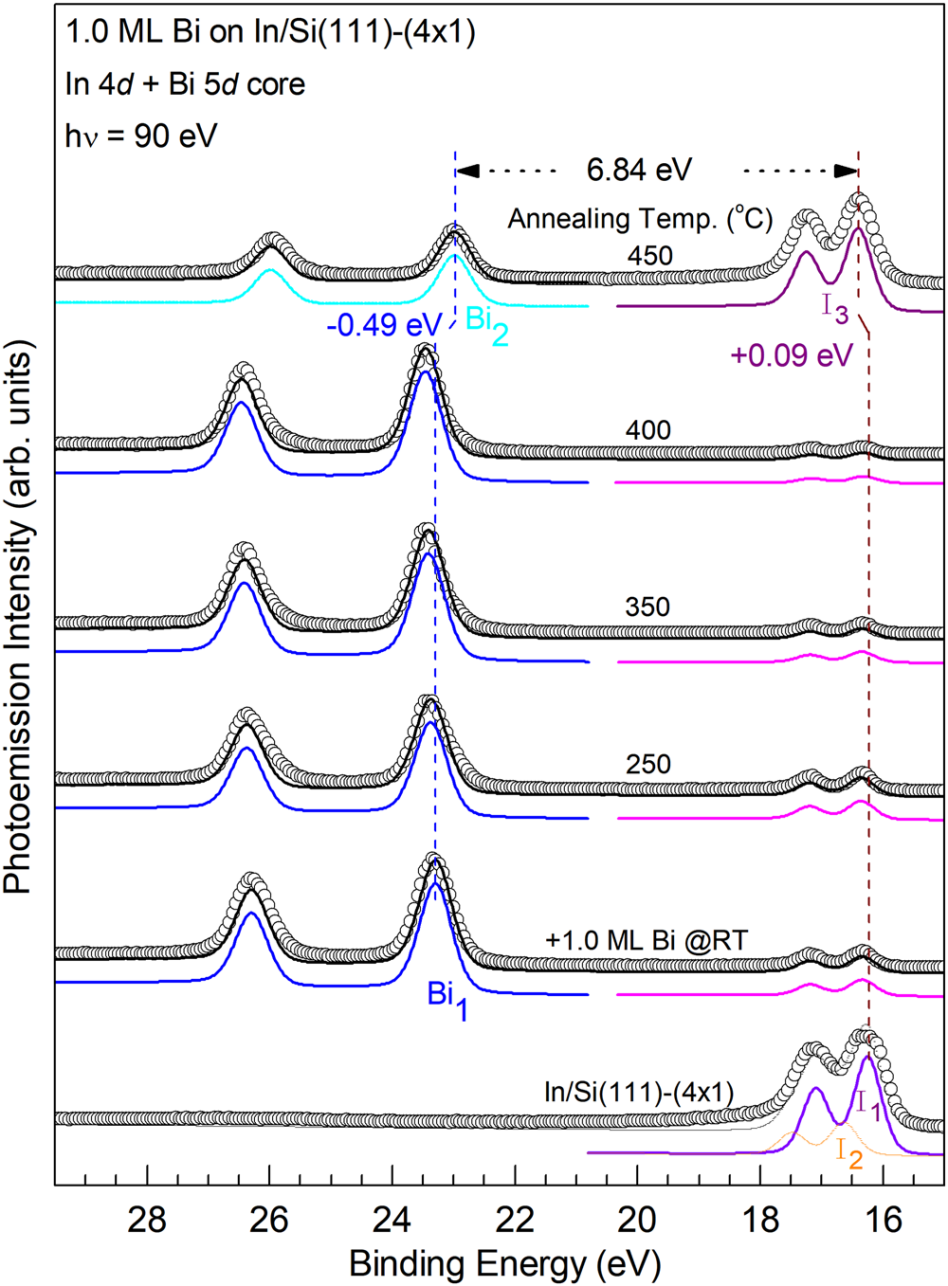
Corresponding Bi 5*d* and In 4*d* core level photoemission spectra(circles) for those in Fig. 3. The black solid curves are results of the least-squares fit with three components Bi_1_ (blue), I_1_(purple) and I_2_(dotted orange). Vertical lines are a guide to the eye. BE Shifts upon 450 °C annealing are indicated.

Figures 3 and 4 show the Si 2*p*, Bi 5*d*, and In 4*d* core level photoemission spectra of the In/Si(111)-(4 × 1) surface (bottom), the same surface after 1.0-ML Bi deposition at RT (second to the bottom) and subsequent annealing at various temperatures as indicated. Only one rather broad component is visually identifiable in all the Si 2*p* core level spectra, suggesting that each of the Si atoms exhibit a similar charging state. The binding energy (BE) of the Si 2*p* shifts to the higher binding energy side by +0.46 eV upon the deposition of 1-ML Bi; this downward band bending of nearly half of the band gap indicates a formation of a dipole layer on the topmost Bi-In layer.

The bottom In 4*d* spectrum in Fig. 4 for the In/Si(111)-(4 × 1) surface can be decomposed into two spin-orbit-doublet components I_1_ and I_2_ with a BE difference of ~0.40 eV. They are attributed to two kind of In atoms on the (4 × 1) surface^41^. Upon deposition of 1.0 ML Bi, the intensity of the In 4*d* core decreases significantly. As will be discussed in the STM section that deposited Bi atoms reside on the topmost layer and no annealing here to provide enough desorption energy, the In 4*d* core electrons exhibit a very small intensity and therefore, suffer from very short escape depth, likely due to both large elastic and inelastic scattering by the topmost Bi layer on their way to vacuum. The bottom Bi 5*d* spectrum shows only one component Bi_1_. As will be further illustrated in the STM observation, the deposited Bi at RT remains on top of In chains; the Bi_1_ component is likely originated from the Bi atoms with the Bi-In bonding. Upon annealing at temperature at ≤400 °C, the line shape and intensities for all the Si 2*p*, In 4*d*, and Bi 5*d* core level spectra remains about the same, suggesting that the Bi/In/Si surface layers retain most of their bonding configurations. At 450 °C, the intensity of the In 4*d* recovers to 67% of that in the bottom spectrum while that of the Bi 5*d* core drops to 46%, suggesting that In atoms move above the Bi layer and the In 4*d* photoelectrons are not strongly scattered. The much reduced intensity of the Bi 5*d* core also indicates of partial Bi desorption from this surface. At the same time, the corresponding BE of the Si 2*p* spectra remains largely the same upon annealing up to ~400 °C and shifts to the lower BE side by −0.40 eV at 450 °C. This revered bend banding (upward) is consistent with fact that In atoms exchanges positions with Bi and becomes the topmost layer.

Scanning tunneling microscopy can obtain real-space atomic-resolved images for the surface and thin films, and thus provide complementary information to chemical compositions and bonding states by the core-level spectroscopy. To clarify the growth and annealing effects of Bi on the 1.0 ML In/Si(111)-(4 × 1) surface shown in the core-level spectra, we have deposited 0.25-ML Bi at RT first. Figure 2(b,c) show the filled-state and empty-state images, respectively. In the images, each of the scattered elongated bright protrusions appear on top of In atoms on one (4 × 1) unit cell on a In/Si(111)-(4 × 1) chain. Many protrusions in the same row are separated by two unit vectors, forming local (4 × 2) ordering. Further deposition of Bi gradually fill each of the In/Si(111)-(4 × 1) chain and the surface consists mainly of the rather ordered (4 × 3) adsorbate reconstruction at 1.0-ML coverage, while some short chains of the (4 × 2) ordering are discernible in Fig. 2(d). These images indicated that the base (4 × 1) In-Si structure is preserved upon the adsorption of Bi at RT and that Bi atoms form ordered bonding on top of the In atoms. The topmost Bi atoms also appear to exhibit a strong attenuation effect for photoelectrons from the In subsurface layers as the In 4*d* photoelectron intensity in Fig. 4 is markedly diminished. Since the silicon atoms in the substrate are deeper than In atoms and Si 2*p* photoelectrons in Fig. 3 are not attenuated strongly, the very short In 4*d* photoelectron escape depth is possibly due both to elastic and inelastic scattering by Bi on top^42^.

The 1.0-ML Bi/In/Si(111)-(4 × 1) surface remains largely the same upon annealing at ≤250 °C. Above 300 °C, the surface gradually becomes disordered. As Fig. 2(f) displays, after annealing at 400 °C, some negative islands of ~2.2 Å deep and local (2 × 2) units appear at the expense of the (4 × 3) and (4 × 2) surface structures. As mentioned in the last section, an annealing at around 450 °C causes a notable change in the core level spectra. The corresponding STM image such as Fig. 2(g) shows that honeycomb cells cover half of the surface. In between the honeycomb chains/areas are similar but disordered protrusions. The surface appears to be very similar to that (2 × 2) found by co-deposition of 0.68 ML of Bi and 1.0-ML In at RT and followed by 250–500 °C annealing^10^, however, the domains with honeycomb cells in Fig. 2(g) are smaller. The spectra of Bi 5*d* and In 4*d* after 450 °C annealing in Fig. 4 indicates that the amount of Bi and In has dropped from 1.0 ML to about 0.50 ML and 0.75 ML, respectively. The Bi/In ratio of 0.66 is close to that reported produced by the co-deposition. Thus, the (2 × 2) structure either from the co-deposition or two-step deposition described in this section are the same: ref.^10^ has proposed a two-atom-layer system with three Bi atoms per (2 × 2) unit cell forming trimer in the top layer and four In atoms in the bottom layer residing on the bulk-like truncated Si(111)-(1 × 1) surface. However, the recovery of the highly attenuated intensity of In 4*d* core level in Fig. 4 suggests that the In atoms move atop of Bi upon 450 °C annealing. The band bending showing in the Si 2*p* core level spectrum upon RT Bi deposition concurrently disappears, supporting the hypothesis that In atoms are on top.

### Growth Processes of In on the β-Bi/Si(111)-(√3 × √3) surface

**Figure 5:**
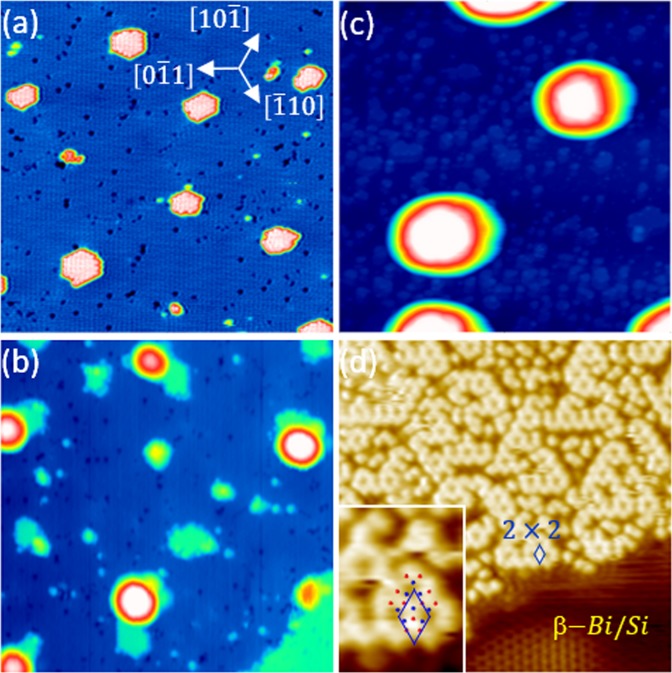
The filled-state STM images of (**a**) the β-Bi/Si(111)-(√3 × √3) surface (β-Bi/Si), the same surface after the deposition of (**b**) 0.2-ML In at RT. (**c**) and (**d**) are obtained after annealing the sample of 1.0-ML In on the β-Bi/Si at 250 and 460 °C. In (**a**), the ad-islands having the same bi-layer step height of Si(111) and the same β-Bi/Si ordering are due to coalesce of adatoms on the Si(111)-(7 × 7). The z-range in (**b**) and (**c**) are 1.8 and 10.3 nm, respectively. V_s_ = −2.5 V; I_t_ = 80 pA. size: (**a**, **b**) 40 × 40 (**c**) 120 × 120; (**d**) 20 × 20 nm^2^. Inset in (**d**): zoom-in STM image superimposed with the atomic model in Fig. 1(b).

In growing the III-V compound films, the polarity affects the crystalline quality and properties, and therefore, controlling the polarity is very important to obtain a desired film. In the previous section, the two-step growth has shown to be different than that found in the co-deposition approach. To investigate the effect of the deposition sequence, we first deposit 1.0 ML Bi of the clean Si(111) surface following by 400 °C annealing for 1.0 min. As Fig. 5 display, this procedure typically produces the β-Bi/Si surface, where 1.0 ML Bi atoms form ordered trimers^27^.

**Figure 6:**
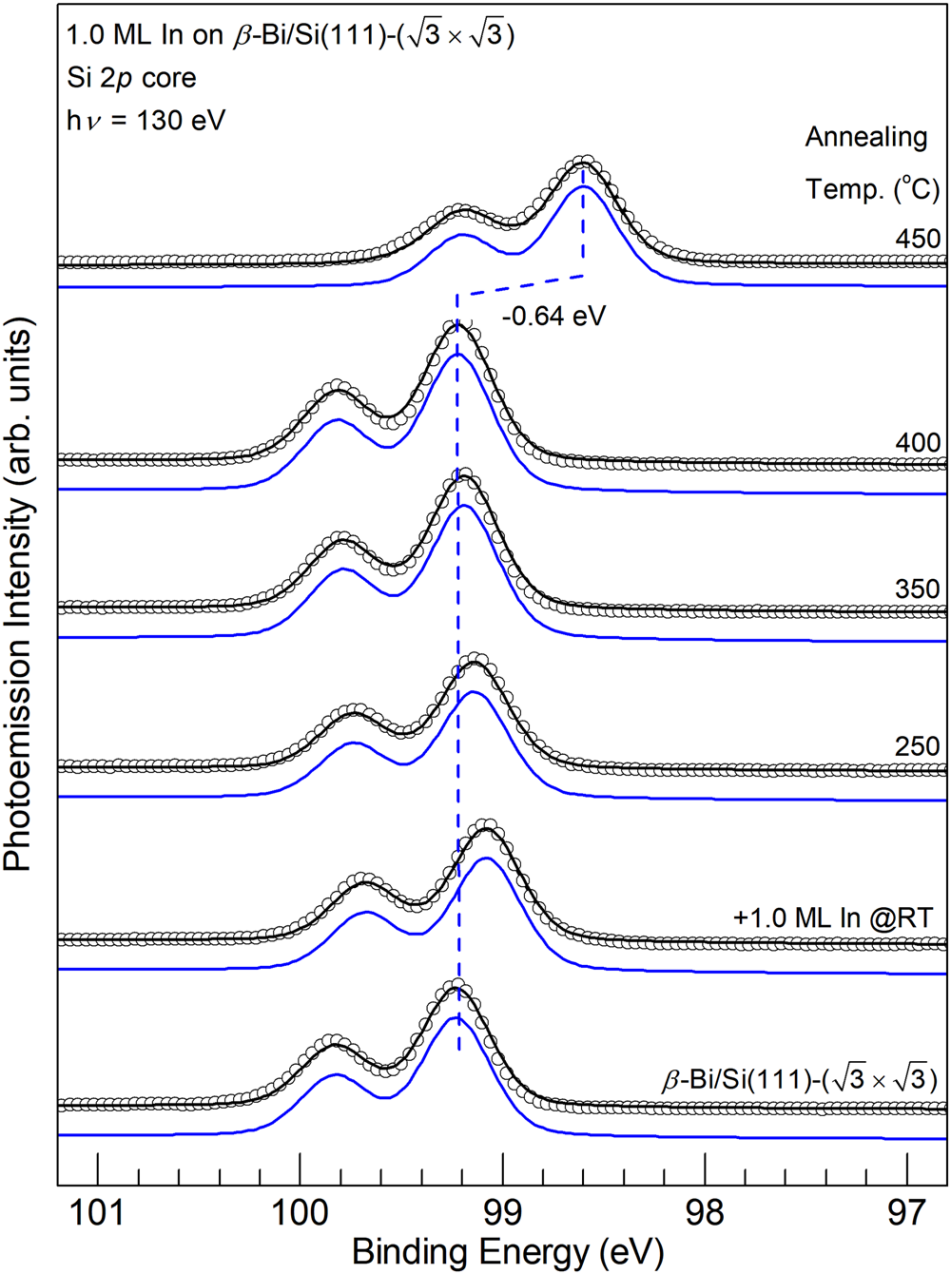
Si 2*p* core level photoemission spectra (circles) of the 1.0 ML β-Bi/Si(111)-(√3 × √3) surface (bottom), the same surface after 1.0-ML of In deposited at RT (second to the bottom) and subsequent annealing at various temperature as indicated. Dashed lines are a guide to the eye.

**Figure 7:**
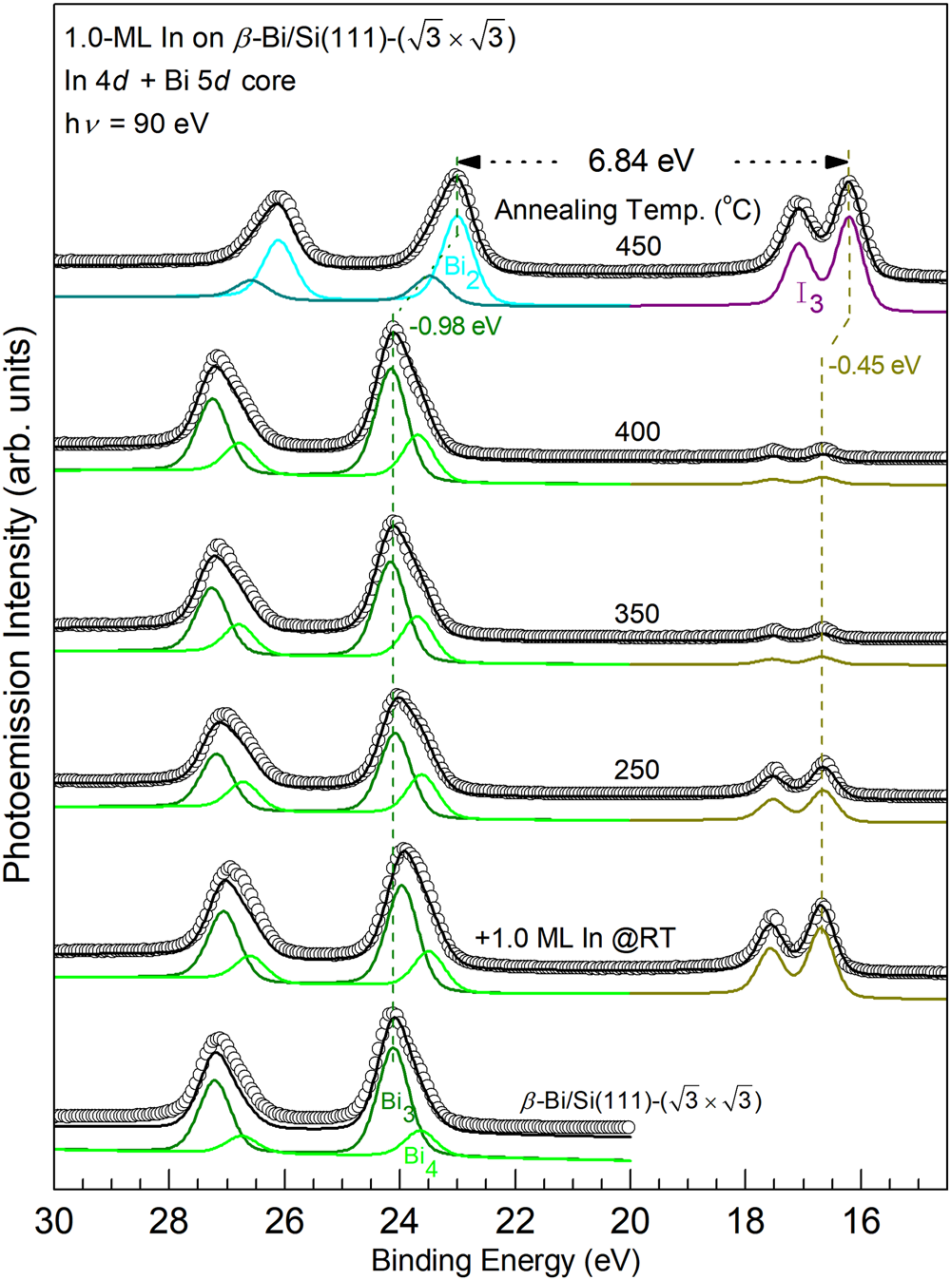
Corresponding Bi 5*d* and In 4*d* core level photoemission spectra for those in Fig. 6. Bi 5*d* and In 4d core level spectra (circles); the black solid curves are results of the least-squares fit with components Bi_2_ (cyan), Bi_3_ (olive), Bi_4_ (green). Vertical dotted lines are guides to the eye. BE Shifts upon 450 °C annealing are indicated.

Figures 5(b,c) show that In atoms from the evaporator apparently nucleate around ad-islands/defects into 3D islands of various sizes and height. The height and base areas of these 3D islands increases in proportion to the amounts of In deposition. At 1.0-ML In coverage after annealing at 250 °C, the average size of the 3D islands is ~300 nm^2^ and the average height is 8.15 nm as shown in Fig. 5(c). The total amount of these In coverage is estimated to be ~1.1 ML on the surface, which is close to the expected value. This observation agrees with the corresponding photoemission analysis in Figs 6 and 7 as discussed in the following.

Figures 6 and 7 show the Si 2*p*, Bi 5*d*, and In 4*d* core level photoemission of the β-Bi/Si surface (bottom), the same surface after 1.0-ML of In deposited at RT (second to the bottom) and subsequent annealing at various temperature as indicated. Again, only one component is visually noticeable in all the Si 2*p* core levels, suggesting that the charge transfer to Si upon the adsorption In and Bi overlayers is small. The binding energy (BE) of the Si 2*p* shifts to the lower binding energy side by −0.16 eV upon the deposition of 1.0 ML In. The BE shifts slightly to higher binding side upon annealing up to ~400 °C and shifts −0.64 eV again to the lower BE side relative to the bottom spectrum at 450 °C. In comparison to that in Fig. 3, this relative large shift in BE suggests an upward band bending by a formation of a dipole layer with In atoms residing on top of the Bi layer.

The bottom Bi 5*d* spectrum in Fig. 7 for the β-Bi/Si(111) surface consists a major spin-orbit-doublet component Bi_3_ and another Bi_4_ component on its shoulder with a lower BE of ~0.46 eV and a smaller intensity of ~1/4. The Bi_3_ component is likely originated from the trimer structure. The origin of the Bi_4_ component is possibly emission from the defects and step edges seen in Fig. 5(a). After the RT growth of 1.0 ML In on the β-Bi/Si surface, the line shape of the Bi 5*d* spectrum remains about the same while its intensity drops by ~20%. Upon annealing the In/Bi/Si surface with increasing temperature, the line shapes of the Bi 5*d* spectra remain about the same while the intensity of the In 4*d* spectra drops by >98% at 400 °C. This observation is consistent with STM observation that the 3D In islands further coalesce into fewer but larger islands (not shown here). At 450 °C, the intensity of In increases more than 100 times while that of Bi 5*d* drops by ~17%. The relative binding energy of the Bi 5*d* and In 4*d* is reduced to be 6.84 eV, which is the same as that seen in Fig. 4. The corresponding STM image shown in Fig. 5(d) is similar (2 × 2) domains as that in Fig. 2(g). The apparent height of the (2 × 2) domains is higher than the β-Bi/Si areas by 2.0 Å, which is close to that found in the DFT calculation (Fig. 1(b)). All data strongly suggest that, upon annealing at ~450 °C, both In/Bi/Si and Bi/In/Si growth sequence lead to the same In-terminated In_0.75_Bi/Si(111)-(2 × 2).

## Conclusions

The polarity of the group III-V compound films of various atomic-thick layers can affect their electronic structures and topological properties and further growth into two and more atomic layers. It is thus preferable to be able to control the polarity of the first overlayer. Synchrotron radiation core-level photoemission spectroscopy, scanning tunneling microscopy (STM), and first-principles calculations have been utilized to explore the growth processes and the atomic structure of the resulting films during the two-step molecular beam epitaxy of In and Bi on the Si(111) surface. These techniques are complimentary and provide high resolution chemical information, atomic-resolved real-space images, and theoretical calculations, respectively. Deposition of 0.5- and 1.0-ML Bi on the In/Si(111)-(4 × 1) surface at room temperature result in Bi-terminated BiIn-(4 × 2) and (4 × 3) structure, respectively. These two structures are stable up to ~300 °C. By contrast, adsorbed In atoms nucleate into 3D islands on the Bi/Si(111)-(√3 × √3)-β surface upon deposition at room temperature. Despite of the different growth modes, annealing at 460 °C results in the same In-terminated In_0.75_Bi/Si(111)-(2 × 2) in the above mentioned two growth sequences. DFT calculation confirms that the surface energy of the A (In-terminated) surface is slightly lower than that of the B (Bi-terminated) surface. The small energy gain could be the reason that the Bi-terminated surface is stable up to ~400 °C. Our findings provides a practical method to control the polarity of the atomic-thick In-Bi film on the Si(111) surface. The two-step MBE growth method is likely applicable to other possible III-V or II-VI compounds with heavier elements.
